# Synergistic Antitumor Effect of Oligogalacturonides and Cisplatin on Human Lung Cancer A549 Cells

**DOI:** 10.3390/ijms19061769

**Published:** 2018-06-14

**Authors:** Cian-Song Huang, Ai-Chun Huang, Ping-Hsiu Huang, Diana Lo, Yuh-Tai Wang, Ming-Chang Wu

**Affiliations:** 1 Department of Food Science, National Pingtung University of Science and Technology, Pingtung 91201, Taiwan; abc340414@gmail.com (C.-S.H.); hugh0530@gmail.com (P.-H.H.); 2 Department of Leisure, Recreation and Tourism Management, Tzu Hui Institute of Technology, Pingtung 92601, Taiwan; justice8j@gmail.com; 3 Department of Food Technology, Bina Nusantara University, Alam Sutera Campus, Alam Sutera, Tangerang 15143, Indonesia; diana.lo@binus.edu; 4 Life Science Center, Hsing Wu University, LinKou District, New Taipei City 24452, Taiwan

**Keywords:** cisplatin, oligogalacturonides, synergistic effect, adjuvant therapy

## Abstract

Cisplatin (DPP), a clinically potent antineoplastic agent, is limited by its severe adverse effects. The aim of this study was to investigate the effect of oligogalacturonides (OGA) and DDP on human lung cancer A549 cells. The combined use of OGA and DDP had a synergistic effect on the growth inhibition of A549 cells, changed the cell cycle distribution, and enhanced apoptotic response, especially in sequential combination treatment group of DDP 12 h + OGA 12 h. Western blot analyses showed that the combination treatment of OGA and DDP upregulated Bax, p53, and Caspase-3 and downregulated Bcl-2 proteins. More importantly, DDP-induced toxicity was attenuated by OGA and DDP combination treatment in normal HEK293 cells. Our data suggests that the combined use of OGA from natural sources and DDP could be an important new adjuvant therapy for lung cancer as well as offer important insights for reducing kidney toxicity of DDP and delaying the development of DDP resistance.

## 1. Introduction

Lung cancer is one of the most common types of fatal malignancies worldwide and was estimated to be the leading cause of cancer deaths in 2016 in the United States [1,2]. The use of adjuvant therapies has not been optimized in lung cancer patients, hence it is expected to develop a broad availability of more targeted therapeutic treatment for lung cancer with limited damage to the immune system and natural barriers [3].

Chemotherapy is usually the main option for cancer patients with advanced stages of neoplasia; however, drug resistance and adverse side effects of chemotherapeutic agents influence the long-term outcomes of patients with advanced stage cancers [4]. Cisplatin, also known as cisplatinum or cis-diamminedichloroplatinum (II), is one of the most effective chemotherapeutic drugs widely used for clinical cancer treatment including urinary bladder, head and neck, lung, ovarian, and testicular cancers. The common cisplatin concentration used for patients is 20–30 mg/m^2^, but in some cases that require a high dosage, the concentration can reach 80–120 mg/m^2^. It has been reported that cisplatin dosage at 50–100 mg/m^2^ may cause nephrotoxicity in 28–36% of patients [5]. The drug resistance and undesirable side effects retard the widespread utility of cisplatin (DDP), hence the combination of cisplatin with other chemical agents or compounds is used for many human cancers to overcome the drug resistance and considerable side effects [6,7].

Evidence suggests that the beneficial effect of high fruit and vegetable intake to several chronic diseases and mortality (mainly cancer) was associated with antioxidant micronutrients, such as vitamin C, provitamin A, and lycopene [8]. Fruit and vegetable intake is associated with a 21% reduced risk of lung cancer risk in women, especially apple intake, which exhibited a significant effect on lung cancer risk decrease in women [9]. The health benefits usually focus on phytochemicals, and the health effect of pectin is mainly as a dietary fiber that exhibits a cholesterol-lowering and gastrointestinal disorder-reducing effect. Pectin derived from tomato, grape, apple, and citrus fruits with protective or disease preventive properties is not well discussed.

The attractive biological activities of pectin and its derivatives include immunostimulating activity, cancer chemoprevention and anti-inflammation, anti-metastatic property, and cancer chemotherapy improvement [10,11,12,13]. Several studies of pectin modified by temperature, pH, and enzymatic process to decrease its molecular weight and degree of esterification have exhibited a potential utilization in pharmaceuticals, nutraceuticals, or diet changes. These studies have included heat-modified citrus pectin, pH-modified citrus pectin, pectinase-hydrolyzed citrus pectin, CPE-II (pectic polysaccharide) form pectinase digestion of citrus peels, and low-molecular-weight citrus pectin [14,15,16].

Oligogalacturonides (OGA) from microbial pectinase hydrolytic citrus pectin were found to express antioxidant activity and lipid oxidation inhibition ability in food emulsion, as well as exhibit a bactericidal effect against foodborne pathogens. OGA is also reported to suppress the proliferation of tumor cells including human hepatoma HepG2, lung carcinoma A549, and colon carcinoma Colo 205 cells, especially the fragments with a molecular weight less than 1 kDa, while untreated pectin showed no significant activity [17,18]. No research was found in the synergistic effect of pectin derivatives and chemotherapeutic drug cisplatin on lung cancer inhibition, nor in the toxicity attenuation of cisplatin. The aim of this study was to investigate the synergistic effect of citrus OGA on cisplatin-based treatment against human lung cancer cells (A549 cells). Additionally, the cytotoxicity reduction of cisplatin on normal HEK293 cells by OGA combination was evaluated to provide a potential therapeutic strategy for human lung cancer treatment.

## 2. Results and Discussion

### 2.1. Effects of OGA and DDP on Cell Proliferation

The growth inhibition of DDP and OGA on human lung cancer A549 cells and normal human embryonic kidney HEK293 cells for a total 24 h of incubation are shown in Table 1. The effects of DDP and OGA on cell viability were assessed by MTT assay. The cell proliferation of HEK293 and A549 cells was inhibited by 52.2% and 21.2%, respectively, at 8 μg/mL DDP, while 9.4% and 12.7% were inhibited, respectively, at 100 μg/mL OGA after 24 h of incubation. It was verified that HEK293 cells were more sensitive to DDP treatment at 2–10 μg/mL compared to that treated by OGA (100 μg/mL). DDP acted more destructively against HEK293 cells than A549 cells in a dose- and time-dependent manner.

In comparison, the treatment between DDP 24 h and DDP + OGA 24 h, combined treatment (DDP + OGA 24 h) resulted in 56.5% (1–26.8/(9.4 + 52.2)) reduction of DDP cytotoxicity on HEK293 cells and a 1.26-fold (42.6/(12.7 + 21.2)) improvement of DDP cytotoxicity on A549 cells at 8 μg/mL. The combined treatment of OGA and DDP also exhibited a synergistic effect in reducing the cell viability of A549 cells at a higher level of DDP (8–10 μg/mL), indicating that OGA might enhance the sensitivity of DDP.

Moreover, the DDP 12 h + OGA 12 h sequential combination treatment expressed the highest and synergistic growth inhibition on A549 cells at 2–10 μg/mL DDP. It resulted in a 2.07-fold (38.3/(7.0 + 11.5)) improvement of DDP cytotoxicity on A549 cells at 6 μg/mL. Meanwhile, the OGA 12 h + DDP 12 h sequential combination treatment expressed a 1.36-fold (25.1/(7.0 + 11.5)) improvement of DDP cytotoxicity on A549 cells at 6 μg/mL. The sequential combination treatment of DDP 12 h + OGA 12 h and OGA 12 h + DDP 12 h resulted in a 37.4% (1–22.4/(6.0 + 29.8)) and 37.7% (1–22.3/(6.0 + 29.8)) reduction of DDP cytotoxicity on HEK293 cells, respectively.

In other words, OGA combined with DDP treatment expressed a synergistic effect on tumor growth inhibition and attenuated the effect of DDP toxicity on normal HEK293 cell lines. All three combination treatments of DDP and OGA reduced the toxic response of DDP on HEK293 cells, indicating that OGA can be used as a protective agent in DDP-induced kidney toxicity. DPP causes renal toxicity through the formation of reactive oxygen species (ROS). By adding OGA after DDP treatment, OGA can neutralize the ROS produced by DDP through its antioxidant activity. By adding OGA before DDP treatment, OGA provides a cytoprotective effect by preventing ROS formation [19].

Moreover, these combined treatments of OGA and DDP exhibited synergistic effects on reducing the cell viability of A549 cells, indicating that combined treatments of OGA and DDP are a valuable option for human lung cancer therapy.

Astolfi et al. [20] indicated that the main factor affecting the severity of adverse effects was the dosage of cisplatin administered. Duan et al. [21] revealed that the appropriate dosing intervals could remarkably delay the development of DDP-resistance. In addition, DDP was found to induce significant renal damage in rats [22]. Therefore, OGA might be a viable adjuvant of DDP chemotherapy. The combined use of OGA and DDP may be a potential strategy for DDP-base adjuvant therapy of human lung cancer. Moreover, OGA might remarkably reduce the kidney toxicity of DDP and delay the development of DDP resistance.

Lactate dehydrogenase (LDH) is a cytosolic enzyme and the release of LDH into a medium indicates the loss of membrane integrity [23]. Hence, LDH activity is a good marker for membrane permeability and cytotoxicity. In order to determine the effect of OGA and DDP on LDH leakage, cells were treated with various combination of OGA and DDP and then LDH leakage was measured. As shown in Table 2, DDP and OGA exhibited cytotoxicity against A549 cells as compared to untreated cells and normal HEK293 cells.

These results revealed that OGA was not only harmless to normal HEK293 cells, but also helpful to reduce LDH leakage from DDP-treated HEK293 cells. A549 cells were more sensitive to the combination treatment of OGA and DDP as compared to OGA or DDP treatment. Cells treated with the combination of OGA and DDP including DDP + OGA 24 h, DDP 12 h + OGA 12 h, and OGA 12 h + DDP 12 h showed significantly higher LDH activity values in the medium than DDP and OGA alone (DDP 24 h or OGA 24 h), indicating that the combination of OGA and DDP were more potent in causing A549 cytotoxicity, especially the DDP 12 h + OGA 12 h sequential combination treatment. As compared to the other combination treatment, DDP 12 h + OGA 12 h sequential combination treatment showed the highest release of LDH activity in media.

Huang et al. [15] revealed that OGA might alter the membrane properties of tumor cells and lead cells to be more susceptible to OGA-induced damage. The main mechanism of DDP is to induce DNA breakage [24]. Therefore, the function of OGA in a synergistic antitumor effect for improving DNA damage caused by DDP in A549 cells might be related to the OGA-induced membrane permeability enhancement.

### 2.2. Morphological Changes of Treated Cells

**Figure 1:**
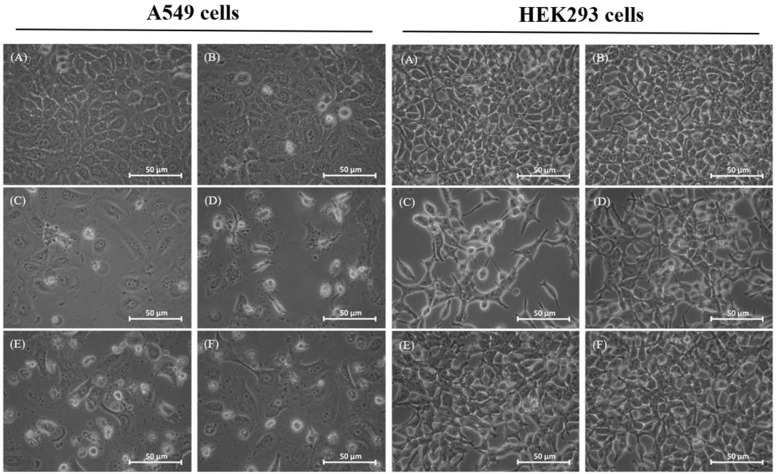
Morphology of DDP- and OGA-treated human lung cancer A549 cells and normal human embryonic kidney HEK293 cells. (**A**) Control, (**B**) OGA 24 h, (**C**) DDP 24 h, (**D**) DDP + OGA 24 h, (**E**) DDP 12 h + OGA 12 h, (**F**) OGA 12 h + DDP 12 h. Control: untreated A549 cells, DDP: cisplatin (2 μg/mL), OGA: oligogalacturonides (100 μg/mL).

The morphological changes of human lung cancer cells A549 caused by DDP and OGA are shown in Figure 1. After DDP treatment, the phenomenon of cell shrinkage occurred, accompanied by rounded cell appearance, poor cell adhesion, and cell number reduction. Comparatively, there was no significant change in the morphology of human embryonic kidney HEK293 cells when treated with the sequential combination of DDP and OGA. Moreover, the combination treatment of DDP and OGA also reduced cell shrinkage and cell number reduction.

**Figure 2:**
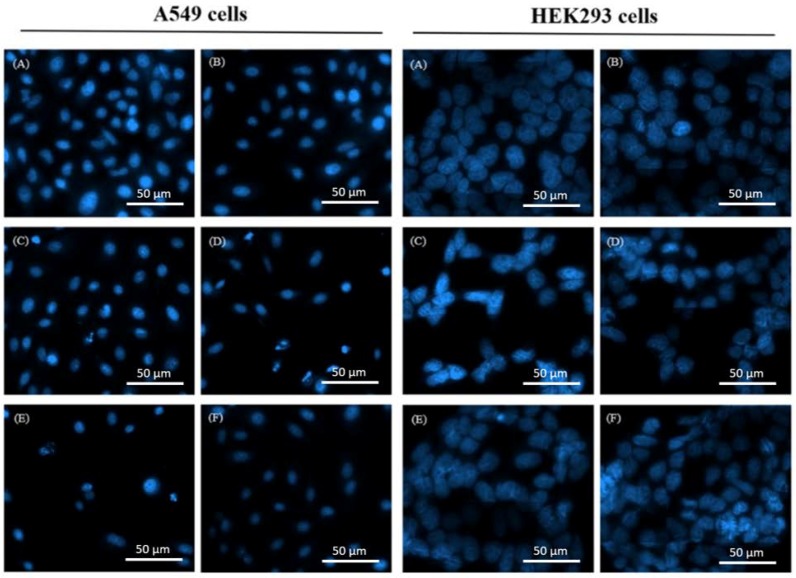
DAPI (4′,6-diamidino-2-phenylindole) fluorescence staining of DDP- and OGA-treated human lung cancer A549 cells and normal human embryonic kidney HEK293 cells. (**A**) Control, (**B**) OGA 24 h, (**C**) DDP 24 h, (**D**) DDP + OGA 24 h, (**E**) DDP 12 h + OGA 12 h, (**F**) OGA 12 h + DDP 12 h. Control: untreated A549 cells, DDP: cisplatin (2 μg/mL), OGA: oligogalacturonides (100 μg/mL).

Furthermore, fluorescence microscopic examination of DAPI (4′,6-diamidino-2-phenylindole)-stained cells was performed to confirm the synergistic effects between OGA and DDP. Chazotte [25] revealed that the fluorescence increased approximately 20-fold when DAPI was bound to double-stranded DNA. Following treatment with 2 µg/mL DDP and 100 µg/mL OGA, the cell number and fluorescence were lower than those of cisplatin- or OGA-treated samples alone (Figure 2). Among them, the DDP 12 h + OGA 12 h sequential combination treatment expressed the lowest cell number and more rounded cell appearance, indicating the existence of a synergistic damage effect of OGA and DDP on the double-stranded DNA of A549 cells, as well as an increased number of apoptotic cells. In contrast to A549 cells, DDP 12 h + OGA 12 h caused no harm to normal human kidney HEK293 cells, as shown from the cell number and the shape of cell.

Zeidan et al. [26] revealed that cisplatin treatment may affect the actin cytoskeleton and induces marked changes in cell morphology. Vassilopoulos et al. [27] proposed that cisplatin treatment plays an inhibitory role in metastasis through blocking cytoskeletal remodeling. Therefore, the synergistic effect of OGA and DDP might also contribute to metastasis prevention.

### 2.3. Combined Effects of OGA and DDP on Cell Cycle Distribution and Apoptosis

**Figure 3:**
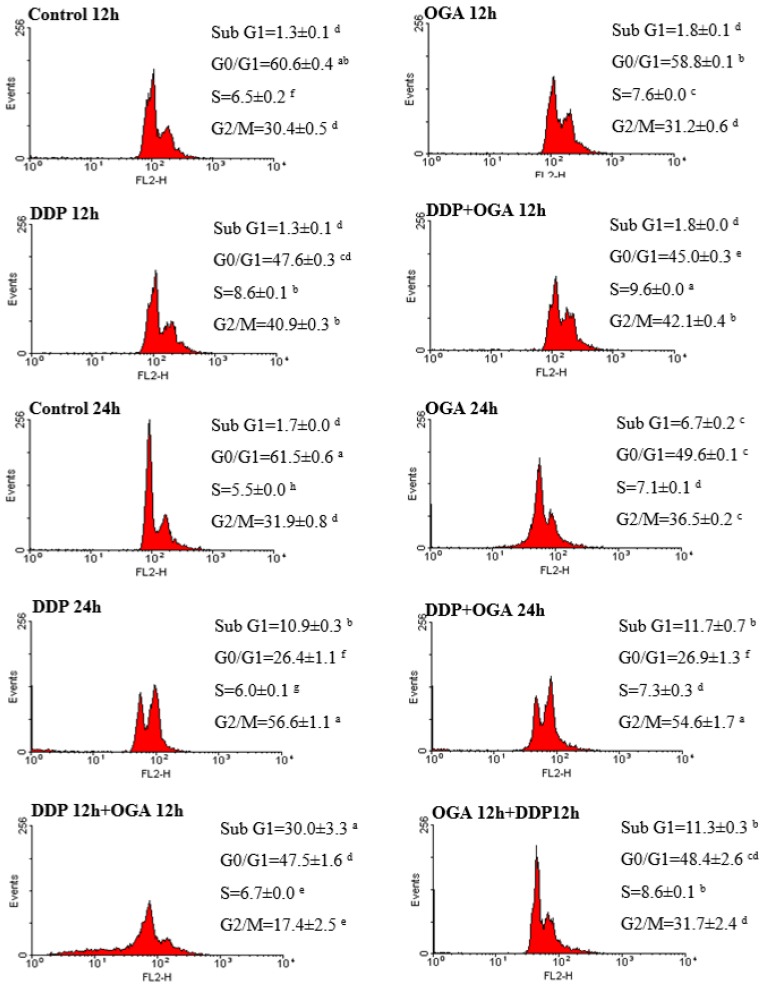
Flow cytometry analysis of cell cycle in A549 cells after DDP and OGA treatment. Cells were cultivated with OGA, DDP, DDP + OGA, DDP then OGA, and OGA then DDP at 37 °C for a total of 12 to 24 h. Values are expressed as means ± SD. Values bearing different letters are significantly different from one another in the same cell cycle population (*p* > 0.05). Control 12 h: untreated and 12 h-incubated A549 cells, Control 24 h: untreated and 24 h-incubated A549 cells, DDP: cisplatin at 2 μg/mL, OGA: oligogalacturonides at 100 μg/mL.

**Figure 4:**
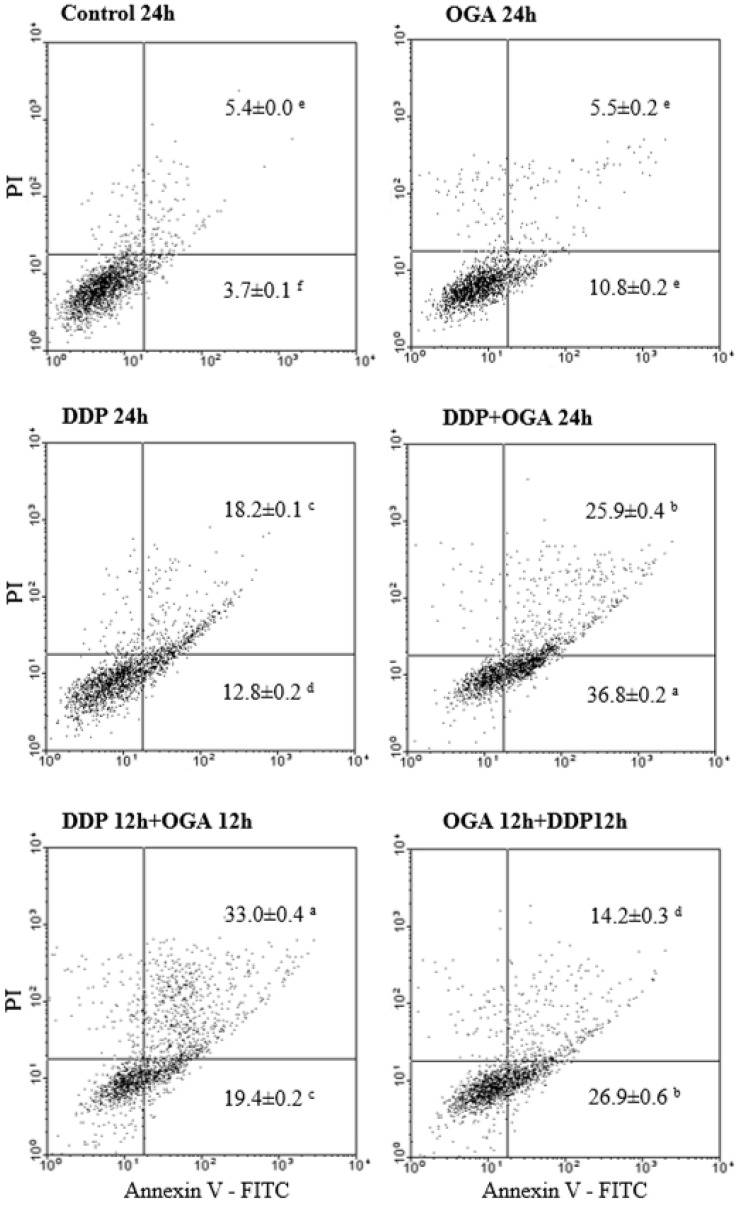
Flow cytometry analysis of apoptosis determination in A549 cells after DDP and OGA treatment. Cells were cultivated with OGA, DDP, DDP + OGA, DDP then OGA, and OGA then DDP at 37 °C for a total of 24 h. Values are expressed as means ± SD. Values bearing different letters are significantly different from one another in the same block position (*p* > 0.05). Control 24 h: untreated and 24 h-incubated A549 cells, DDP: cisplatin at 2 μg/mL, OGA: oligogalacturonides at 100 μg/mL.

Flow cytometry analysis was carried out to evaluate the effect of OGA and DDP on cell cycle distribution (Figure 3) and apoptosis determination (Figure 4) in A549 cells. The histograms of cell cycle distribution indicated that OGA or DDP treatment could suppress cell proliferation, increased the sub G1 fraction, and accumulated cells in the G2/M phase at 37 °C for a total of 24 h. The combined use of OGA and DDP changed the cell cycle distribution and enhanced the apoptotic response in the sub G1 fraction, especially in the DDP 12 h + OGA 12 h group. Moreover, the accumulation of cells was shifted to the G0/G1 phase. The cell population of the DDP + OGA 24 h treatment was similar than that of the DDP 24 h treatment. However, the effect of the sequential combination treatments (DDP 12 h + OGA 12 h and OGA 12 h + DDP 12 h) on A549 cells showed a remarkable cell cycle specificity. The DDP 12 h + OGA 12 h sequential combination treatment showed a greater cell cycle change than that in DDP 24 h, OGA 24 h, and DDP + OGA 24 h treatments.

In OGA-enhanced cisplatin-mediated apoptosis, A549 cells were more sensitive to OGA treatment when pretreated with DDP. Further incubating the cells treated with DDP for 12 h and then OGA for 12 h (DDP 12 h + OGA 12 h treatment) led to an increased sub G1 phase. The fraction of A549 cells in the G0/G1 phase of the cell cycle decreased from 61.5 to 47.5% after DDP 12 h + OGA 12 h treatment. By contrast, the fraction of cells in the G2/M phase of the cell cycle decreased from 31.9 to 17.4%. The apoptotic death was confirmed by flow cytometry using Annexin V-FITC (Figure 4). Annexin V-FITC was used to determine the percentage of cells that endured early and late apoptosis. The results revealed that DDP 12 h + OGA 12 h treatment had most of cells going through late apoptosis (33.0%), while DDP + OGA 24 h treatment caused most of cells to go through early apoptosis (36.8%). This finding supported the LDH released data, which also indicated the occurrence of late apoptosis. 

Liu et al. [28] revealed that A549 cells were blocked in the G1 phase after treatment with cisplatin. Interestingly, the cell cycle of cisplatin-treated A549 cells was arrested at the G2/M phase and changed to the G0/G1 phase under further OGA treatment in the present study. An increase in the sub G1 population indicated an increase in apoptotic cells, hence treating A549 cells with DDP and OGA might amplify the apoptotic cascade. Moreover, the sequential combination treatment with OGA then DDP showed a different cell population as compared to the other two combination treatments, indicating that the apoptotic pathways of DDP then OGA and OGA then DDP sequential combinations and the DDP + OGA combination treatment might be different. Further investigation of the synergistic effect of native antitumor components on DDP-induced apoptotic pathways might give additional insights into the anti-tumor mechanisms of combination therapy.

### 2.4. Mechanistic Studies of OGA-DDP Synergism

**Figure 5:**
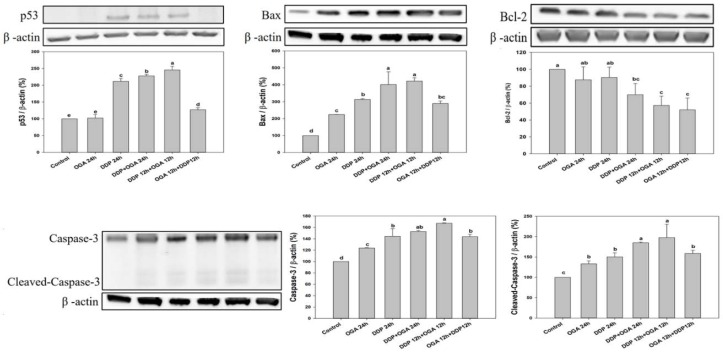
Apoptosis effect of OGA and DDP on human lung cancer cells (A549). A549 cells were treated with 100 μg/mL of OGA or 2 μg/mL of DDP at 37 °C for 24 h, followed by Western blot analysis. p53, Bax, Bcl-2, Caspase-3, and Cleaved-Caspase-3 were examined by Western blot analysis. Values are expressed as means ± SD of three measurements. Means bearing the same superscript small letters in the same row are not significantly different (*p* > 0.05). DDP: cisplatin (2 μg/mL), OGA: oligogalacturonides (100 μg/mL).

The mechanisms underlying the synergism between OGA and DDP were examined using Western blot analysis of A549 cells after a total of 24 h of treatment. OGA and DDP upregulated Bax, Caspase-3, and Cleaved-Caspase-3. The combined treatment of OGA and DDP upregulated p53, Bax, Caspase-3, and Cleaved-Caspase-3 and decreased Bcl-2 proteins (Figure 5). OGA-treated A549 cells exhibited cell cycle arrest in the absence of p53 expression according the expression levels. However, the combination treatment of OGA and DDP upregulated the expression of p53.

**Figure 6:**
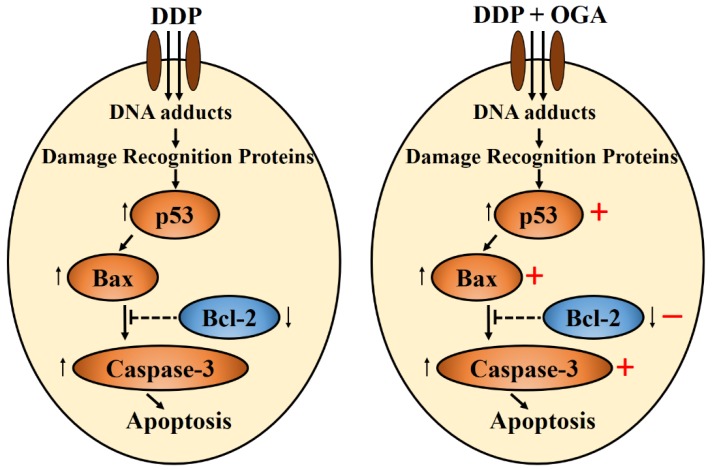
Schematic diagram of the synergistic effect of OGA on DDP-induced apoptosis in human lung cancer A549 cells. DDP: cisplatin, OGA: oligogalacturonides.

Low-molecular-weight citrus pectin was reported to downregulate Bcl-xL and Cyclin B to promote apoptosis [16]. Vermes and Haanen [29] stated that the tumor suppressor gene p53 regulates the cell response to DNA damage and cell progression. Zamble et al. [30] indicated that cisplatin inhibits cell proliferation by two possible mechanisms, p53-dependent apoptosis and p53-independent cell cycle arrest. In the present study, OGA was found to upregulate Bax and Caspase-3 to promote apoptosis via a p53-independent pathway. The apoptosis of DDP-treated A549 cells was enhanced when combined with OGA, and the combination treatment of OGA and DDP upregulated p53, indicating that the combined use of OGA and DDP amplified the p53-dependent mechanism. OGA enhanced DDP-induced apoptosis in human lung cancer A549 cells via a p53-independent caspase-3-dependent pathway (Figure 6). Further investigation of other non-apoptotic signaling pathways can help in better understanding of the anti-tumor mechanisms of combination therapy.

## 3. Materials and Methods

### 3.1. Materials

Commercial microbial pectic enzyme Peclyve CP containing 133.5 U/mL pectin lyase (PL) activity, 50.6 U/mL pectin methyl esterase (PME) activity, and 22.4 U/mL polygalacturonase (PG) activity from *Aspergillus niger*, was purchased from Lallemand Australia Pty. Ltd. (North Adelaide, Australia). Citrus pectin with a 60% degree of esterification (DE) was purchased from Nacalai Tesque (Kyoto, Japan). Fetal bovine serum (FBS) and Trypsin-EDTA solution (10×) were purchased from Biological industries (Kibbutz Beit Haemek, Israel). Dullbecco’s Modified Eagle’s medium (DMEM), Kaighn’s Modification of Ham’s F-12 Medium, Thiazolyl Blue Tetrazolium Bromide (MTT), 4′,6-diamidino-2-phenylindole (DAPI), and cisplatin (DDP) were purchased from Sigma-Aldrich (St. Louis, MO, USA). Lactate dehydrogenase (LDH) cytotoxicity detection kit was purchased from Takara Bio (Shiga, Japan). Annexin V apoptosis detection kit FITC was purchased from eBioscience (San Diego, CA, USA). All chemicals used in this study were analytically pure and of culture grade. The primary antibodies against Bax (#5023S), Bcl-2 (#4223S), p53 (2524S), and Caspase-3 (#9662S) were purchased from Cell Signaling Technology, Inc. (Beverly, MA, USA). The primary antibodies against β-Actin (#J2114) were purchased from Santa Cruz Biotechnology (Santa Cruz, CA, USA). Fluorochrome-conjugated Goat anti-mouse lgG (#AP124P) and Goat anti-rabbit lgG antibodies (#AP132P) were purchased from Merck Millipore (Billerica, MA, USA). Bio-Rad Protein Assay Dye Reagent Concentrate, Laemmli sample buffer, and 2-Mercaptoethanol were purchased from Bio-Rad (Hercules, CA, USA).

### 3.2. Preparation of Oligogalacturonides

Oligogalacturonides (OGA) with molecular weight <1 kDa and zero degrees of esterification (DE) were produced according to Huang et al. [17] with some modification. In brief, OGA was obtained by the enzymatic degradation of 100 mL citrus pectin (1% *w*/*v*) with 5 mL commercial microbial pectic enzyme (containing 0.13 U/mL PL activity, 0.05 U/mL PME activity, and 0.02 U/mL PG activity) at pH 4, 45 °C for 48 h. After inactivation, the mixture of OGA was freeze-dried and the DE of OGA was confirmed to be zero. The molecular weight of OGA (48 h enzyme-treated pectin) is less than 1 kDa, consisting of 2.93% arabinose, 12.64% mannose, 5.15% galactose, and 51.09% glucose [31].

### 3.3. Cell Culture and Treatments

Human lung carcinoma A549 cells (BCRC 60074, Bioresource Collection and Research Center, Hsinchu, Taiwan) were cultured in F12K medium containing 10% (*v*/*v*) FBS. Human normal embryonic kidney HEK293 cells (BCRC 60019, Bioresource Collection and Research Center) were cultured in DMEM containing 10% (*v*/*v*) FBS. All cells were incubated in Petri dishes (Corning, Lowell, MA, USA) and maintained in an exponential phase of growth at 37 °C in a 5% CO_2_-containing humidified incubator. The treatments were divided into single treatment (OGA 12 h, DDP 12 h, OGA 24 h, and DDP 24 h), combined non-sequential treatments (DDP + OGA 12 h and DDP + OGA 24 h), and combined sequential treatments (DDP 12 h + OGA 12 h and OGA 12 h + DDP 12 h). For the combined sequential treatments, DDP (2–10 μg/mL) or OGA (100 μg/mL) were added into cells and, after 12 h incubation, the cultivated solution was removed and changed into OGA (100 μg/mL) or DDP (2–10 μg/mL) and incubated for another 12 h. The DDP concentration used in this study was 2–10 μg/mL, which represents about 15.5–77.6 mg/m^2^ of DDP dosage in patients.

### 3.4. Cell Viability Assay

Growth inhibition of human cells was measured using MTT assay following the methods done by Mosmann [32]. Briefly, cells were cultured in a 96-well plate at an initial concentration of 1 × 10^4^ cells/well in 100 μL medium for 24 h prior to treatment. The culture medium was replaced by fresh medium containing DDP and/or OGA at various concentrations. After incubation and removing the cultivated solution, 100 μL of MTT solution (3 mg MTT/mL phosphate-buffered saline (PBS, 8 g NaCl/1.15 g Na_2_HPO_4_/0.2 g KH_2_PO_4_/0.2 g KCl/l)) was added for another incubation. The MTT-formazan product was extracted and its absorbance was measured at 570 nm using an Epoch™ Microplate Spectrophotometer (BIO-TEK^®^, Winooski, VT, USA). The growth inhibition of DDP and OGA on A549 and HEK293 cells was calculated according to the following formula:

Growth inhibition (%) = [1 − (absorbance of treatment/absorbance of control)] × 100%.

### 3.5. Cell Morphology Assay

Cells were seeded in a 6-well plate (1 × 10^5^ cells/well) and cultured overnight for cell adhesion. The culture medium was then removed and replaced with DDP (2 μg/mL) and OGA (100 μg/mL) containing medium for a further 12 to 24 h of incubation, and the morphological changes of cells were observed under a microscope (Primovert, Zeiss, Gottingen, Germany). Fluorescent staining analysis was carried out following culture and treatments as previously described, after which cells were seeded on glass coverslips in a 6-well plate and then fixed in 4% paraformaldehyde for 1 h, permeabilized with 0.3% Triton X-100 for 5 min and blocked with 1% BSA for 15 min at room temperature. The cells were stained with 1 mmol/L DAPI for 1 h at 4 °C in a dark room and detected under a fluorescence microscope (TissueGnostics, TissueFAXS and HistoFAXS, TissueGnostics, Wien, Austria).

### 3.6. Detection of Lactate Dehydrogenase (LDH) Activity

LDH activity was measured using an LDH cytotoxicity detection kit, with some modification. Briefly, cells (1 × 10^4^ cells/well) were seeded in a 96-well plate at 37 °C for 24 h and then cultivated with a 200-μL sample for 24 h. Subsequently, the medium (100 μL/well) was transferred into a fresh 96-well plate for LDH activity determination as described in the kit manual. The activity of released LDH (expressed as the absorbance at 490 nm) in the medium was measured and calculated as follows:

LDH activity = (absorbance of sample − absorbance of control)/(absorbance of

triton X-100 treated − absorbance of control) × 100%

### 3.7. Cell Cycle Analysis

A549 human lung cancer cells (1 × 10^5^ cells/well) were seeded in a 6-well plate, incubated at 37 °C for 24 h, and treated with DDP- and OGA-containing medium. After incubation, cells were harvested, washed with PBS, and fixed in 70% ethanol. Subsequently, cells were centrifuged, washed, re-suspended in 1 mL PBS containing 10 μg/mL RNase A and 20 μg/mL propidium iodide (PI), followed by 30 min of incubation in the dark [33]. Cell cycle analysis was performed on a FACScan flow cytometer (Becton Dickinson, Franklin Lakes, NJ, USA) with 10,000 cells counting/sample and the cell cycle phase was determined by WinMDI version 2.9 (Scripps Research Institute, La Jolla, CA, USA).

### 3.8. Detection of Apoptosis by Annexin V-FITC

Cellular apoptosis was analyzed using an Annexin V apoptosis detection kit FITC. A549 human lung cancer cells (1 × 10^5^ cells/well) were seeded in a 6-well plate and treated with DDP- and OGA-containing medium. After 24 h, cells were digested with trypsin, washed with PBS three times, suspended in 500 μL of binding buffer, and then mixed with 5 μL of FITC-conjugated Annexin-V and 5 μL of PI and incubated for 15 min at room temperature in the dark. Apoptosis determination was performed on a FACScan flow cytometer (Becton Dickinson, Franklin Lakes, NJ, USA) with 10,000 cells counting/sample and the apoptosis phase was determined by WinMDI version 2.9 (Scripps Research Institute, La Jolla, CA, USA).

### 3.9. Western Blot Assay

After the abovementioned treatments, cells were washed three times with PBS and lysed in RIPA buffer (25 mM Tris/HCl pH 7.6, 150 mM NaCl, 1% Nonidet-P40, 1% Sodium deoxycholate, 0.1% SDS) with EDTA (100:1) and protease inhibitor cocktail (100:1) on ice for 20 min. The lysates were then centrifuged at 12,000× *g* for 10 min, and supernatants were collected. Protein concentrations were determined by Bio-Rad Protein Assay Dye Reagent Concentrate. The proteins (60 μg) were denatured in 2-Mercaptoethanol containing Laemmli sample buffer and separated by sodium dodecyl sulfate-polyacrylamide gel electrophoresis (SDS-PAGE), and then transferred onto a polyvinylidene difluoride membrane (Amersham, Germany). The membrane was incubated with the appropriate primary antibody (diluted 1:1000), fluorochrome-conjugated secondary antibody (diluted 1:5000), and visualized by enhanced chemiluminescence according to the manufacturer’s instructions (Millipore, Billerica, MA, USA) in the chemiluminescent detection system (ECL, Thermo Scientific, Barrington, IL, USA). The results were analyzed with the Image J software (National Institutes of Health, Benthesda, MD, USA) to determine the relative ratio. β-actin was used as a loading control. The value of the control was set as 100.

### 3.10. Statistical Analysis

All measurements were carried out in three independent replications. Statistical analysis was performed using SPSS Version 16 (SPSS Inc., Chicago, IL, USA). Statistical comparisons were made by one-way analysis of variance (ANOVA) followed by Duncan’s multiple range test. Values were considered significantly different when *p* < 0.05.

## 4. Conclusions

OGA in combination with DDP exhibits a synergistic antitumor effect in A549 cells and attenuates DDP toxicity on normal HEK293 cell lines, especially in the DDP 12 h + OGA 12 h sequential combination treatment. The maintenance of DDP-sensitive cells through appropriate treatment intervals may help to reduce the DDP concentration needed and control the development of DDP resistance. Therefore, OGA has a high potential as a novel therapeutic strategy to improve the clinical therapy of lung cancer. The combined use of OGA maybe of promising therapeutic value for lung cancer and provide important insights for reducing the kidney toxicity of DDP and delaying the development of DDP resistance; thus, this treatment warrants further investigation.

## Figures and Tables

**Table 1:** Growth inhibition of cisplatin (DDP) and oligogalacturonides (OGA) on human lung cancer A549 cells and normal human embryonic kidney HEK293 cells.

**(A) Growth Inhibition (%) on A549 Cells**						
**Treatment**	**DDP (** **μg/mL)**					
	**0**	**2**	**4**	**6**	**8**	**10**
OGA 12 h	7.0 ± 1.2	-	-	-	-	-
DDP 12 h	-	8.1 ± 1.7 ^cE^	8.9 ± 1.0 ^cE^	11.5 ± 2.6 ^cD^	19.5 ± 2.6 ^bC^	34.1 ± 2.5 ^aD^
DDP + OGA 12 h	-	11.2 ± 0.8 ^cDE^	14.1 ± 2.9 ^cD^	20.7 ± 3.8 ^bBC^	38.3 ± 1.6 ^aB^	41.5 ± 1.7 ^aC^
OGA 24 h	12.7 ± 1.0	-	-	-	-	-
DDP 24 h	-	13.8 ± 2.8 ^cCD^	15.1 ± 2.5 ^cCD^	16.8 ± 3.6 ^bcC^	21.2 ± 2.3 ^bC^	39.8 ± 2.2 ^aC^
DDP + OGA 24 h	-	17.0 ± 1.0 ^dBC^	25.3 ± 2.9 ^cB^	22.3 ± 2.9 ^cB^	42.6 ± 1.2 ^bA^	61.5 ± 1.7 ^aA^
DDP 12 h + OGA 12 h	-	26.8 ± 3.1 ^dA^	30.5 ± 3.2 ^dA^	38.3 ± 1.2 ^cA^	45.5 ± 0.4 ^bA^	53.5 ± 1.2 ^aB^
OGA 12 h + DDP12 h	-	18.0 ± 2.1 ^bcB^	19.1 ± 0.5 ^bcC^	25.1 ± 3.0 ^aB^	22.4 ± 2.9 ^abC^	15.3 ± 3.0 ^cE^
**(B) Growth inhibition (%) on HEK293 cells**						
**Treatment**	**DDP (μg/mL)**					
	**0**	**2**	**4**	**6**	**8**	**10**
OGA 12 h	6.0 ± 2.6	-	-	-	-	-
DDP 12 h	-	23.7 ± 2.8 ^cC^	28.3 ± 2.7 ^bcB^	29.8 ± 4.2 ^bB^	31.3 ± 2.4 ^abB^	35.4 ± 1.3 ^aC^
DDP + OGA 12 h	-	12.2 ± 2.5 ^dE^	17.8 ± 1.8 ^cC^	19.5 ± 3.7 ^cC^	24.1 ± 1.6 ^bC^	28.8 ± 0.7 ^aE^
OGA 24 h	9.4 ± 1.2	-	-	-	-	-
DDP 24 h	-	37.9 ± 0.5 ^dA^	43.2 ± 0.8 ^cA^	51.5 ± 2.7 ^bA^	52.2 ± 3.1 ^bA^	57.9 ± 1.0 ^aA^
DDP + OGA 24 h	-	27.1 ± 1.3 ^bB^	27.2 ± 0.6 ^bB^	24.1 ± 2.3 ^bC^	26.8 ± 3.3 ^bC^	37.8 ± 1.1 ^aB^
DDP 12 h + OGA 12 h	-	16.8 ± 2.1 ^cD^	21.8 ± 5.0 ^bC^	22.4 ± 1.0 ^bC^	32.4 ± 1.3 ^aB^	33.3 ± 1.0 ^aD^
OGA 12 h + DDP12 h	-	11.4 ± 0.8 ^dE^	19.9 ± 4.0 ^cC^	22.3 ± 1.1 ^cC^	27.1 ± 1.5 ^bC^	32.1 ± 1.2 ^aD^

Cells were cultivated with OGA, DDP, DDP + OGA, DDP then OGA, and OGA then DDP at 37 °C for a total of 12 to 24 h. Values are expressed as means ± SD of three measurements. Means bearing the same superscript capital letters in the same column, and the same superscript small letters in the same row are not significantly different (*p* > 0.05). OGA: oligogalacturonides at 100 μg/mL.

**Table 2:** Cytotoxicity of DDP and OGA on human A549 cancer cells.

Treatment	LDH (Lactate Dehydrogenase) Activity (%)	
	A549	HEK293
Control 12 h	0.0 ± 0.1 ^g^	0.0 ± 0.2 ^f^
OGA 12 h	4.3 ± 0.3 ^f^	0.2 ± 0.7 ^f^
DDP 12 h	11.2 ± 4.0 ^e^	14.5 ± 2.5 ^b^
DDP + OGA 12 h	15.6 ± 1.1 ^d^	5.9 ± 0.1 ^d^
Control 24 h	0.0 ± 1.0 ^g^	0.0 ± 0.1 ^f^
OGA 24 h	23.1 ± 2.6 ^c^	0.4 ± 0.0 ^f^
DDP 24 h	26.9 ± 4.3 ^c^	26.6 ± 2.6 ^a^
DDP + OGA 24 h	36.1 ± 2.3 ^b^	11.2 ± 0.3 ^c^
DDP 12 h + OGA 12 h	49.4 ± 2.2 ^a^	3.5 ± 0.9 ^e^
OGA 12 h + DDP12 h	35.2 ± 1.9 ^b^	3.1 ± 0.1 ^e^

Cells were cultivated with OGA, DDP, DDP + OGA, DDP then OGA, and OGA then DDP at 37 °C for a total of 12 to 24 h. Values are expressed as means ± SD of three measurements. Means bearing the same superscript small letters in the same column are not significantly different (*p* > 0.05). Control 12 h: untreated and 12 h-incubated A549 cells, Control 24 h: untreated and 24 h-incubated A549 cells, DDP: cisplatin at 2 μg/mL, OGA: oligogalacturonides at 100 μg/mL.
